# Sensory Processing Impairments in Children with Developmental Coordination Disorder

**DOI:** 10.3390/children9101443

**Published:** 2022-09-22

**Authors:** Huynh-Truc Tran, Yao-Chuen Li, Hung-Yu Lin, Shin-Da Lee, Pei-Jung Wang

**Affiliations:** 1 Department of Physical Therapy, Graduate Institute of Rehabilitation Science, China Medical University, Taichung 40402, Taiwan; 2 Department of Occupational Therapy, Asia University, Taichung 41354, Taiwan; 3 Department of Physical Therapy, Asia University, Taichung 41354, Taiwan

**Keywords:** developmental coordination disorder, sensory processing, motor coordination, sensory integration, sensorimotor

## Abstract

The two objectives of this systematic review were to examine the following: (1) the difference in sensory processing areas (auditory, visual, vestibular, touch, proprioceptive, and multi-sensory) between children with and without developmental coordination disorder (DCD), and (2) the relationship between sensory processing and motor coordination in DCD. The following databases were comprehensively searched for relevant articles: PubMed, Science Direct, Web of Science, and Cochrane library. There were 1107 articles (published year = 2010 to 2021) found in the initial search. Full-text articles of all possibly relevant citations were obtained and inspected for suitability by two authors. The outcome measures were sensory processing impairments and their relationship with motor coordination. A total of 10 articles met the inclusion criteria. Children with DCD showed significant impairments in visual integration, tactile integration, proprioceptive integration, auditory integration, vestibular integration, and oral integration processes when compared with typically developing children. Evidence also supported that sensory processing impairments were associated with poor motor coordination in DCD. Preliminary support indicated that DCD have sensory processing impairments in visual, tactile, proprioceptive, auditory, and vestibular areas, which might contribute to participation restriction in motor activities. It is important to apply sensory integration therapy in rehabilitation programs for DCD in order to facilitate participation in daily activities.

## 1. Introduction

Developmental coordination disorder (DCD) is a neurodevelopmental disorder, and its proportion is roughly 5–6% in children aged five to eleven years old [1,2]. According to the Diagnostic and Statistical Manual of Mental Disorders, fifth edition (DSM-V), children with DCD are characterized by poor motor coordination when compared with age-matched children with typical development [3]. The deficiency in motor coordination occurs in the early developmental period, and children with DCD have shown participation restrictions in self-care, leisure, and physical activities.

Sensory processing is referred to as the capacity to manage the detection, modulation, interpretation, and organization of incoming sensory cues [4]. In Dunn’s model, sensory processing patterns are described as the interplay between people’s neurological thresholds and behavioral responses from self-regulation strategies [5]. From this view, there are four sensory processing patterns: low registration (high threshold and passive self-regulation), seeker (high threshold and active self-regulation), sensor (low threshold and passive self-regulation), and avoider (low threshold and active self-regulation). Furthermore, sensory processing patterns deeply influence a child’s behavior. A sizeable variation in the degree and pattern of perceptual–motor deficits has been shown in DCD children [3]. Some earlier literature demonstrated that deficiencies were found in visual–motor integration [6,7,8], impaired visual sensitivity [9], or visual–spatial processing in children with motor coordination problems. However, inconsistent findings regarding visual–perceptual ability have been found by previous studies. One study found that DCD children showed impaired visual sensitivity when differentiating children with typical development, whereas the other study showed no significant group difference [6,9]. In terms of tactile discrimination [10], two studies reported that children with DCD showed more deficits in proprioceptive processing function than children with typical development, such as the localization of single and double simultaneous stimuli, graphesthesia, fingers identification [11,12], and two-point discrimination in both moving and static conditions [10]. The above reviews indicated that children with poor motor coordination experienced predominately perceptual issues, but further study is required in order to examine the characteristics of visual or tactile processing function in children with DCD. Proprioceptive information not only affects the detection and correction of movement errors and the regulation of coordinated movement, but also influences individual participation in daily life [13,14]. Previous studies have reported that the considerable impairment in proprioceptive processing that leads to motor coordination problems has been found in DCD [15,16,17]. However, the empirical results with regard to proprioceptive abnormalities in children with DCD are inconsistent. Several studies have reported that DCD children have more impairments in proprioceptive processing function than typically developing children [16,17,18], while other studies reported that there was no association between proprioceptive dysfunction and motor coordination problems in DCD [19,20,21,22].

The below existing evidence indicates that coordination deficit and sensory processing difficulty are primary obstacles in DCD children. Misaki et al. (2018) found that abnormal sensory processing function might cause the pathophysiology of DCD [23]. Another study also reported that there were some difficulties in sensory processing and sensory integration in children with DCD, which may, in turn, hinder these children from participating in routine activities [24]. Poor sensorimotor integration is one of the most implicated causes of motor coordination challenges in DCD children. Wilson et al. (2017) indicated that diminished white matter organization in sensorimotor structures varied the transmittance of structural connectivity in the whole brain network in children with DCD; this could affect their motor skills [25]. Zwicker JG et al. (2012) demonstrated that decreased axial diffusivity in the motor and sensory tracts was the key cause of DCD [26]. Imaging studies reported that the left superior and inferior parietal lobules were less activated in children with DCD while doing continuous and visuomotor tracking tasks [27]. The above literature has shown that there is a possibility of a relationship between sensory processing function and motor coordination competence in children with DCD; this needs further study in order to explore and interpret the underlying mechanism and design efficient intervention approaches for DCD children.

Therefore, the two aims of this study were to systematically review the literature and to better understand (1) the difference in sensory processing areas (auditory, visual, vestibular, touch, proprioceptive, and multi-sensory) between children with and without DCD, and (2) the relationship between sensory processing function and motor coordination competence in DCD.

## 2. Materials and Methods

### 2.1. Search Strategy

The methodology used in the study was based on the guidelines from the Cochrane Handbook [28]. The protocol of this systematic review was previously published in the PROSPERO database, and the registration number is CRD42021249197. A comprehensive search for sensory processing functions that have been used to measure sensory processing was administrated within multiple computerized databases, including PubMed, Science Direct, Web of Science, and Cochrane library. The following Medical Subject Headings (MeSHs) were used: (“sensory” OR “sensorimotor” OR “sensory processing” OR “sensory deficit”) AND (“developmental coordination disorder” OR “DCD” OR “coordination disorder” OR “clumsy” OR “dyspraxia”). In addition, we also read references from SELECTED studies to find other eligible papers.

### 2.2. Inclusion Criteria

The following were the criteria for a study to be included: (1) The study population should be children with DCD. (2) The studies aimed to inspect both the sensory processing impairment and its relationship with motor coordination. That is, a standardized measure or scale for assessing the sensory processing function or motor coordination competence was used. (3) A control group of typically developing (TD) children was recruited. (4) The full text article was written in English and was published between 2010 to 2021.

### 2.3. Exclusion Criteria

Papers were excluded if participants with DCD had other comorbidity medical diagnoses, such as autism spectrum disorder or attention-deficit/hyperactivity disorder.

### 2.4. Outcome Targets

The studies aimed to examine both the sensory processing impairment and its relationship with motor coordination. A standardized measure or scale for assessing the sensory processing function or motor coordination competence needed to be used by the studies.

### 2.5. Data Extraction and Analysis

The Newcastle–Ottawa Quality (NOQ) Assessment Scale [29] was utilized to access the methodological quality of each study based on the suggestions of the Cochrane Non- Randomized Studies Methods Working Group for case-control and cohort studies. The nine criteria of the NOQ were composed of three key domains: (1) selection of study groups (four criteria), (2) comparability of study groups (one criterion), and (3) ascertainment of the outcome of interest (three criteria). This scale utilized a star rating system for each study with a total score ranging from 0 to 9 stars. More stars indicated a higher study quality. Two authors (Hung-Yu Lin and Pei-Jung Wang) evaluated the quality assessment independently, with discrepancies resolved by the other author (Yao-Chuen Li) (Table 1).

## 3. Results

**Figure 1:**
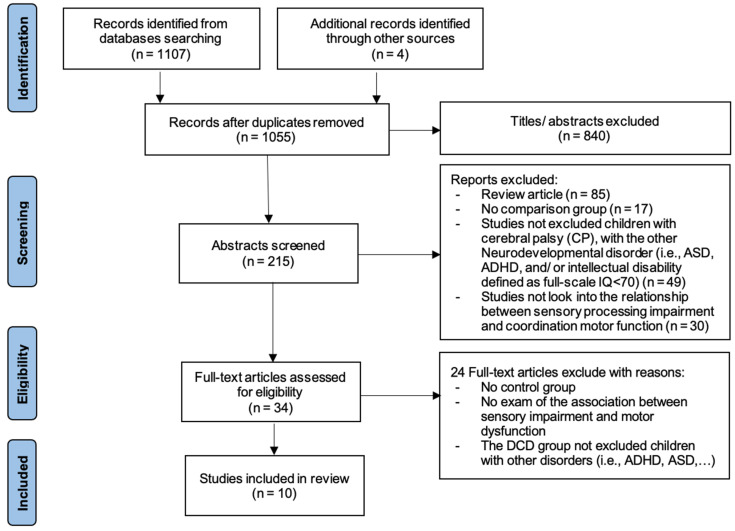
This is a figure. Schemes follow the same formatting.

Figure 1 shows the entire process of searching and selecting the studies for inclusion in this systematic review. A total of 1107 studies were found in the databases, and 4 studies were found in the additional records identified through other sources from the initial search. After accounting for duplicates, we reviewed the titles and abstracts of 1055 articles; 840 articles were excluded based on our inclusion criteria. Most of the studies demonstrated topics not equivalent to the subject of our review. We reviewed the full text of the remaining 34 articles, and considered a total of 10 articles that fitted the inclusion criteria. The reasons for excluding the 24 full-text articles were no control group; the DCD group not excluding children with other disorders such as ADHD, dyspraxia, and intellectual disability; and no examination of the association between sensory impairment and motor dysfunction.

All 10 articles were cross-sectional studies. Table 2 outlines the study characteristics. The age range of participants were from 5 to 14 years. Different outcome measures were conducted to assess sensory processing dysfunction, including sensory profile (two studies), visual–tactile temporal order judgment task (one study), haptic detection/discrimination task (one study), the developmental test of visual–motor integration (one study), the test of visual perceptual skills (two studies), Semmes Weinstein Monofilaments: Registration (one study), single-point localization (one study), two-point discrimination (one study), Ayres Southern California Sensory Integration Test, and Doing study’s design task (two studies).

**Figure 2:**
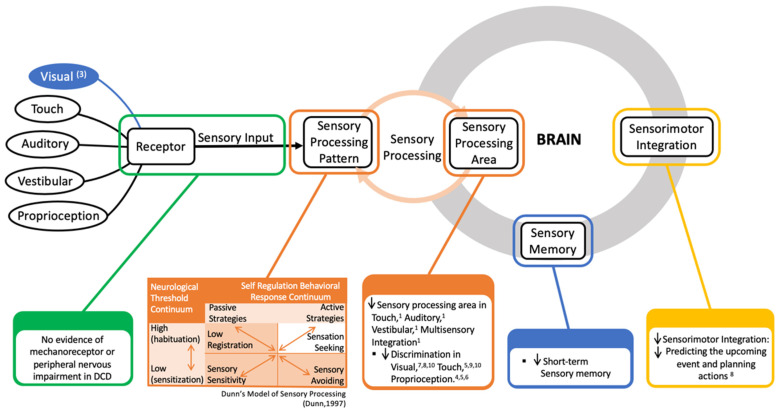
Summary diagram of the sensory deficit in children with developmental coordination disorder; (1), (3)–(10): indicated the study in Table 2 show information given in the diagram. Dunn’s Model of Sensory Processing [30].

Regarding sensory processing, this systematic review categorized them into six processes: visual integration process, touch integration process, proprioceptive integration process, auditory integration process, vestibular integration process, and others. Four studies examined the difference in the impairment of the visual integration process between DCD children and children developing typically. All of the studies indicated a significantly lower visual integration process in DCD. Four studies demonstrated that DCD children had a significantly lower touch integration process score than their TD peers. In addition, two studies found that children with DCD had difficulty in the proprioceptive integration process when compared with the TD group. Two studies reported impairments of the auditory integration process and the vestibular integration process in children with DCD, separately. Figure 2 represents an overview of the sensory deficit in children with DCD.

Figure 2 shows that there is no evidence of a mechanoreceptor or peripheral nervous abnormal to explain proprioceptive impairment in DCD. Previous studies have found impairments in sensory integration in children with DCD. Regarding the sensory processing pattern, they have a lower registration (i.e., hypo-responsiveness to sensory stimuli) and more sensory sensitivity and sensation avoiding compared with TD children. Furthermore, dysfunctional processes of sensorimotor processing (motor planning or motor execution) also accounted as underlying for the sensory deficit in DCD. And reducing short-term sensory memory was found in children with DCD that also contribute to the sensory impairment in DCD. Moreover, compare with the other sensory area, DCD tend to reply on visual sense (having visual bias). 

**Figure 3:**
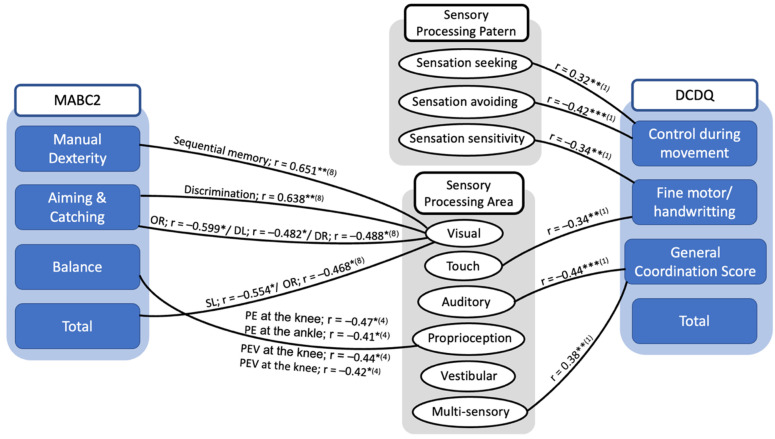
Summary diagram of the correlation between sensory processing and motor coordination in children with DCD. * *p* < 0.05, ** *p* < 0.01, *** *p* < 0.001; OR: double off in the right; DL: double left; DR: double right; SL: single in left in the sequential coupling of eye and hand. In this test, a yellow indication and three feasible target circles with the identical diameter of 10 mm were demonstrated on the screen at the left and right side of the midline individually, and the yellow indication was at the far left and far right locations. Three circumstances with two sides (left or right) were considered: single (one target), double (two targets), and double-off (two targets appeared, then disappeared) (abbreviated as SL, SR, DL, DR, OL, and OR, sequentially). (1); (4); (8): indicated the study in Table 3 showed information given in the diagram.

Table 3 presents the findings of the associations between sensory processing impairment and motor coordination. Two articles explored the relationship by using the Developmental Coordination Disorder Questionnaire—DCDQ. Six studies reported the association of sensory processing and motor coordination measured with the Movement Assessment Battery for Children test—MABC. One study used Jensen–Taylor Test of Hand Function [31] and the Evaluation Tool of Children’s Handwriting [32] and one used the Functional Independence Measure for Children. Figure 3 represents the overview of the correlation between sensory deficit and motor function in DCD children. The significant correlations between sensory processing and the subscale scores on the DCDQ were shown in this study, and two studies indicated a significant correlation between sensory processing area and the MABC-2 total and subscale scores.

## 4. Discussion

To the best of our knowledge, this is the first systematic review to assess sensory impairment (auditory, vestibular, visual, touch, proprioceptive, and multi-sensory) in children with DCD when compared with children developing typically. According to previous reviews, it was conspicuous that the number of children with DCD was comparatively high, but the study findings for the sensory deficit were narrow. In a limited number of studies, minimal studies allocated in some parameters of sensory integration were associated with motor obstacles. Additionally, the studies we included aimed to find the connection between sensory integration deficits and motor coordination problems in DCD children who did not have comorbidity with ASD and ADHD.

### 4.1. Evidence Synthesis for Sensory Impairment in DCD Compared with Non-DCD

Regarding the touch integration process, children with DCD showed more deficiencies in somatosensory. Touch atypicalness might be a prevailing, but less recognized, feature in children with DCD. Registration and perception are two phases composed of tactile function [33]. A tactile stimulus is detected initially and essentially through the registration or sensitivity phase—the forerunner to perception. Subsequently, the sensory input, based on the spatial, temporal, and modality-specific components of a stimulus, is interpreted and gives meaning in the tactile perception or acuity phase, i.e., the smallest perceivable difference between two recognizable curves [34,35]. Therefore, it is crucial to assess both phases to determine the level and severity of the tactile dysfunction [33]. Major studies have found that the magnitude of the diminishing touch acuity was impaired in children with DCD, but no study investigated the haptic sensitivity impairment in DCD. The other quantitative study demonstrated that the difficulty in the sensory integration of touch information was one of the problems in DCD [36]. An alternative study indicated that children with DCD showed an elevated threshold for tactile-related sense, e.g., light touch [37].

Regarding our systematic review, two studies investigated the impairment of proprioceptive in DCD: one allocated the proprioceptive in the upper limbs, and the other in the lower limbs. Both studies found that joint position sense acuity was reduced, indicating proprioceptive deficits in children with DCD. This means that they had a decreased ability to identify joint position through proprioception. Moreover, there is extensive evidence that children with DCD showed greater proprioceptive impairment in distal joints than proximal joints; and this result corroborates previous studies reporting on patients with stroke and intracranial disorders [38,39]. An innovation study using a Sensory profile indicated that lower registration (i.e., hypo-responsiveness to sensory stimuli) was shown in DCD [23,40]; which regarding proprioceptive stimuli detection difficulty.

Regarding the visual integration process, DCD children revealed a substantially more inferior performance compared with TD. Before starting the examination, Nobusako et al. (2021) used a simple stimulus test to demonstrate that visual function was typical in DCD children. An earlier investigation measuring visual evoked potentials indicated that the performance of DCD was similar to TD [41]. The four studies related to visual abilities included in this review mainly estimated the diverse aspects of the visual–perceptual capabilities in DCD. The major findings were that there was a notable decrease in accuracy of the visual–perceptual performance in DCD children compared with those with TD. For visual sensitivity tasks, children with DCD showed a remarkably low sensitivity compared with typical development children. The above finding indicates that DCD had a significantly higher threshold than TD. In signal processing, DCD children tended to rely on visual bias more than the TD children.

Only one study indicated impairment in the auditory, vestibular, and oral processing in children with DCD. In fact, there are various previous experiments that corroborate this finding. The auditory and vestibular sensory integration difficulties, using parent-reporting questionnaires, were found in DCD children from 5 to 12 years old in Allen and Casey’s study [24]. Furthermore, DCD children in the early child period could have trouble with oral movement, for example, eating difficulties and speech/language challenges, because of the oral sensory processing problems [42,43,44].

Fundamentally, Figure 2 represents an overview of the sensory deficits in children with DCD according to our reviews. When the stimuli reach the body in children with DCD, sensory detection—the amounts of sensory stimuli or sensory threshold, needed by a person for recognizing and responding—decreases in the touch and proprioceptive areas, but is normal in the visual, auditory, and vestibular areas. This means that children with DCD need a higher threshold to detect the stimuli in touch and proprioceptive perception. The stimuli, after that, enter the brain for analysis, which is usually called sensory integration or sensory process. Here, almost all visual, touch, proprioceptive, auditory, and vestibular integration encounter problems; this sensory processing difficulty (SPD) has also been proven in some DCD phenotypes in earlier studies [24,45,46]. They displayed a decline in sensory discrimination—the ability to recognize various aspects of stimuli and to distinguish one sensory experience from another—in all sensory senses. Maybe this makes them inaccurate at identifying the differences in stimulus. In addition to the problem of sensory integration, DCD children did not actively reply to the sensory stimuli, which was demonstrated by an increase in sensory sensitivity and low registration. In the same manner, they tended to avoid the sensory input because they felt it much more intensely than the children developing typically did. Children with DCD were also overwhelmed by sensory input, so they showed an increase in sensation avoiding. As a result, DCD children struggled to perform motor coordination challenges because of difficulties in sensory process/integration.

### 4.2. Evidence Synthesis for the Relationship between Motor Performance and Sensory Processing in DCD

There was an association between low thresholds in sensory processing (sensory avoiding and sensory sensitivity) and fine and gross motor problems, and low registration associated with control during movement, general coordination, and the DCDQ-ES total score [23]. Allen and Casey also determined low registration issues in DCD, which was related to approximately 24–33% body awareness and balance difficulties in this population [24]. The association between DCD and passive self-regulation strategies was supported by the above finding. Thus, it was demonstrated that these children might not attempt to respond actively to complexities in stimuli perception.

The association between impairment in tactile integration and fine motor coordination problems in children with DCD was also revealed. Tseng et al., 2019, demonstrated that higher haptic discrimination thresholds were correlated with lower three MABC-2 sub-scores. This indicates that children with higher haptic discrimination thresholds have a tendency to show poorer motor coordination. This finding was similar to a recent study that investigated the correlation between tactile and motor function in children with and without DCD at elementary school [47]. The execution and feedback movement of the upper limb, after receiving stimulus, could be affected by a decrease in tactile spatial perception, proposing that tactile function plays an important role in handwriting proficiency. As a result, inaccurate errors in higher-order processing in the form of spatial tactile perception might contribute to the upper limb coordination problems experienced in DCD children.

Our reviews indicated a connection between proprioceptive deficit and motor in DCD. The severity of proprioceptive deficits in knee and ankle joints was negatively associated with balance performance in children with DCD [48]. This implies that the proprioceptive status in the lower extremities predicted the balance ability in DCD. Proprioceptive JND thresholds were negatively correlated with the manual dexterity score in MABC-2, indicating that children’s wrists with higher thresholds of proprioceptive might have poorer fine motor control [49]. Similarly, upper limb proprioceptive status could serve as a predictor of lower limb balance function, as JND wrist thresholds were also strongly correlated with the MABC-2 balance score.

We also found the tendency that a large number of DCD children showed an increase in visual bias, which might be correlated with their decreased manual dexterity. In the present systematic reviews, children with DCD were illustrated to perform poorer in tasks such as static visual discrimination, visual sequential memory, and sequential coupling of eye and hand. These would cause a decline in the speed of achievement of MABC-2 or daily tasks. According to the mentioned findings, children with DCD could have difficulties in integrating visual signals to accomplish motor duties. Therefore, when evaluating motor functions, it is necessary to carefully assess visual–perceptual ability.

Another finding of one study demonstrated the relationship between auditory processing difficulties and inadequate motor coordination, suggesting that auditory processing (sensory) dysfunction and motor coordination problems originated from the equivalent underlying neural mechanism that involved the cerebellum. Further study would be required to explore the underlying neural mechanism of the association between auditory processing and motor coordination in toddlers with DCD.

### 4.3. Explain the Underlying Mechanism of the Sensory Impairment

The abnormality of the proprioceptive mechanism underpinning DCD is poorly understood, and no study revealed the identification of a distinct neural signature of DCD [50,51]. Notably, several studies found that DCD children had a tendency to show hypotonia [52,53]. With low muscle tone, the length of the skeletal muscle fiber is longer than normal when the muscle is relaxed and/or during a sustained voluntary contraction. Here, the internal force generated by muscle contraction and position or movement of body limbs may not be sufficient to trigger muscle spindle activation. Therefore, this resulted in a low firing rate of proprioceptive afferents, which could further lead to joint position sense deficits in children with DCD. Moreover, one review pointed out a significant association between motor coordination performance and joint hypermobility/joint hyperlaxity [54]. The excessive joint mobility demonstrated a significantly poorer joint proprioception [55] because of the repetitive stresses on joints. The repeated stress on joints damaged receptors, and it diminished joint proprioceptor activation following from capsular or ligamentous stretching [56]. Additionally, as mentioned previously, a higher tactile threshold could account for a reduction in proprioceptive performance in the joints for children with DCD. Correspondingly, dystonia and Parkinson’s disease, which affect the cortico-basal ganglia–thalamo–cortical circuitry, detected similar insufficiency in the proprioceptive acuity [57]. Maybe this mechanism is influenced in children with DCD. Thus, the significant difference found in proprioceptive acuity between joints may explain the possible lesions in certain afferent pathways and/or atypical activations in the proprioceptive-processing neural area in children with DCD [48]. Future research should systematically investigate the possible causes of impaired proprioception in children with DCD in the peripheral nervous system, e.g., muscle spindle sensitivity, the total number of joint mechanoreceptors, intrafusal, and chain fibers. More studies are required to provide possible mechanisms of proprioception difficulties in children with DCD caused by the central nervous system (neural activity in the premotor cortex, cerebellum, and the proprioceptive regions of the basal ganglia and grey matter volume in the precentral gyri, postcentral gyri, insula, and angular gyri). In addition, another remarkable finding was that compared with children developing typically, children with DCD showed a low registration regarding proprioceptive stimuli detection. Low registration issues frequently include challenges in proprioceptive stimuli. Proprioceptive stimuli detection is important for body awareness and balance, and it involved the conscious and subconscious awareness of spatial and kinesthetic parameters of the musculoskeletal framework [58].

The action system is intimately connected with visual perception concerns, including both object recognition and determination in space. This is why if there is any failure in this processing network, it can influence the restriction in movement planning, correction, and feedback [8]. Bonifacci highlighted a demonstrated lower visual–motor processing capacity, but uninfluenced perceptual skills in children at risk for DCD [6]. However, the visual feedback process in DCD is managed differently and much more slowly compared with normal children, according to recent research [59,60]. Maybe atypical brain function leads children with DCD to tend to have visual bias. Evidently, they activate more visual cortex areas during accomplishment tasks when using functional magnetic resonance imaging (fMRI) to evaluate the brain activity [61]. This indicates that DCD children depend more on visual feedback when performing tasks. Similarly, diminishing parietal cortex activity while increasing the activity in the visual cortex, compared with peers [27], also indicates that children with DCD mainly depended on their visual sense to achieve tasks [27,61]. Furthermore, DCD revealed a decrease in the inferior parietal lobule [62], which involves visual and tactile integration [63] and is associated with manual dexterity. Accordingly, the moderate association between increased visual bias in DCD and reduced manual dexterity may be correlated with decreased activity in the inferior parietal region.

Finally, the abnormality in the cerebellum may be responsible for underlying mechanisms of motor coordination problems and sensory processing difficulties in children with DCD, especially auditory sensory [64,65]. Besides, the normal action of the cerebellum involves making motor automatic, abnormality in the cerebellum gives a possible explanation of why children with DCD tended to passively counter with the stimuli. However, the mechanism accounting for oral process problems in children with DCD remains unclear.

## 5. Conclusions

The 10 studies analyzed in the current review reported the general sensory deficits and the link between sensory processing and motor coordination difficulties in DCD. Our reviews also illustrated that the pathophysiology of children with DCD involved an abnormality of sensory integration, and suggest the importance of assessing sensory processing functions. The problems were mainly due to inappropriate methods of integrating information from visual, tactile, proprioceptive, auditory, vestibular, and oral senses. The above challenges caused restriction in the participation of DCD children in daily activities. Therefore, we also recommend adding sensory integration therapy into rehabilitation programs in order to facilitate DCD children’s developmental competence and participation in daily activities.

## Figures and Tables

**Table 1:** Results of risk of bias assessment of the selected studies are based on the Newcastle—Ottawa quality assessment scale.

STUDY	Selection				Comparability		Exposure			Score/Stars
	1	2	3	4	1a	1b	1	2	3	
1. Mikami et al., 2021 [23]	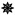	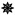	-	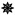	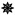	-	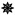	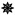	-	6/9
2. Delgado-Lobete et al., 2020 [30]	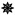	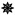	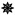	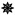	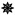	-	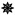	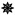	-	7/9
3. Nobusako et al., 2021 [31]	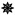	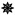	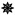	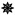	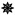	-	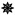	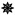	-	7/9
4. Chen et al., 2020 [32]	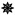	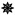	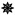	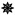	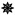	-	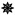	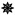	-	7/9
5. Tseng et al., 2019 [33]	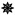	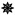	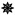	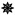	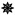	-	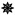	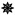	-	7/9
6. Tseng et al., 2018 [34]	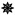	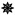	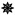	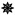	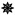	-	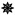	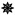	-	7/9
7. Prunty et al., 2016 [35]	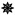	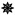	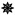	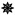	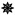	-	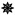	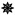	-	7/9
8. Cheng et al., 2014 [36]	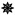	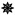	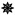	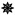	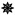	-	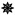	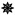	-	7/9
9. Cox et al., 2015 [37]	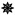	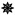	-	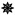	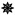	-	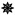	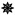	-	6/9
10. Elbasan et al., 2012 [11]	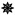	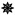	-	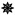	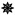	-	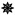	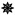	-	6/9

A score with a range of 0–9 was allocated to each study, and those with a score of 6 or more were considered to be high-quality studies.

**Table 2:** Sensory processing impairment in children with and without developmental coordination disorder (DCD).

Study Design (Author, year) Population (Age: M ± SD)	Measures	Sensory Processing Impairment: DCD Group vs. TD or Other Disability Group					
		Touch	Proprioceptive	Visual	Auditory	Vestibular	Appendixes
1. CS (Mikami et al., 2021) [23] 63 DCD (64 ± 2 months) 106 TD (64 ± 2 months)	- Sensory Profile	↓—DCD is poorer	-	-	↓—DCD is poorer	↓—DCD is poorer	↓—DCD is poorer - oral sensory (↑ DCD significant higher in the score of ** oral sensory) ↑ DCD significant higher in the score of ** low registration, ** sensory sensitivity, ** sensation avoiding
2. CS (Delgado-Lobete et al., 2020) [30] 46 DCD (10 ± 2 years) 369 TD (9 ± 2 years)	- Short Sensory Profile-2	-	-	-	-	-	↑ DCD significant higher in the score of * low registration, * sensory sensitivity, * sensation avoiding, * seeking
3. CS (Nobusako et al., 2021) [31] 19 DCD (9.3 ± 1.4 years) 19 TD (9.3 ± 1.4 years)	- Visual–tactile TOJ task	↓—DCD is poorer (↑* DCD significant higher in the score of PSE)	-	-	-	-	-
4. CS (Chen et al., 2020) [32] 28 DCD (10.86 ± 1.07 years) 28 TD (10.96 ± 1.18 years)	- Doing study’s design task	-	↓—DCD is poorer (↑* DCD significant higher in the score of PE at knee; ↑** DCD significant higher in the score of PE at ankle; ↑** DCD significant higher in the score of PEV at ankle)	-	-	-	-
5. CS (Tseng et al., 2019) [33] 20 DCD (10.55 ± 0.72 years) 20 TD (10.65 ± 0.45 years)	- Haptic detection/discrimination task	↓—DCD is poorer (↑*** DCD significant higher in the score of DT)	↓—DCD is poorer (↑*** DCD significant higher in the score of DT)	-	-	-	-
6. CS (Tseng et al., 2018) [34] 20 DCD (10 years 4 months ± 3 months) 30 TD (10 years 5 months ± 3 months)	- Doing study’s design task	-	↓—DCD is poorer (↑* DCD significant higher in the score of SDPE at the wrist; ↑*** DCD significant higher in the score of JND at the wrist)	-	-	-	-
7. CS (Prunty et al., 2016) [35] 28 DCD (10.61 ± 2.23 years) 28 TD (10.95 ± 2.12 years)	- The Developmental Test of Visual–Motor Integration (VMI) - The Test of Visual Perceptual Skills (TVPS)	-	-	↓—DCD is poorer (↓DCD significant lower in the score of *** TVPS; *** VD; * VM; * SR; ** SM; *** VF; ** VC)	-	-	-
8. CS (Cheng et al., 2014) [36] 17 DCD (7.25 ± 0.28 years) 17 TD (7.09 ± 0.34 years)	- Test of Vis-ual Percep-tual Skills-Revised (TVPS-R)	-	-	↓—DCD is poorer (↓ DCD significant lower in the score of * VD; * FC; *** TVPS; * SM; ** VF)	-	-	-
9. CS (Cox et al., 2015) [37] 20 DCD (8.35 ± 1.63 years) 16 TD (8.69 ± 2.24 years)	- Semmes Weinstein Monofilaments: Registration - Single Point Localization (SPL) and two-point discrimination (2PD)	↓—DCD is poorer (↓ DCD significant lower in the score of * SPL—nondominant)	-	-	-	-	-
10. CS (Elbasan et al., 2012) [11] 37 DCD (boys: 10 ± 1.5 years; girls: 10 ± 2 years) 35 TD (boys: 10 ± 0.8 years; girls: 9 ± 1 years)	- Ayres Southern California Sensory Integration Test	↓—DCD is poorer (↓ DCD significant lower in the score of * MFP; * FI; * Graphesthesia; * LTS; * DTSP; * Kinesthesia)	-	↓—DCD is poorer (↓ DCD significant lower in the score of * SV; *** Position in space; ** Design copying)	-	-	-

DCD = developmental coordination disorder; TD = typically developing. ↑: increase sensory processing problem, ↓: decrease sensory processing problem, * *p* < 0.05, ** *p* < 0.01, *** *p* < 0.001; PSE: (point of subjective equality) is a quantitative indicator of perceptual bias; PE: position error; PEV: position error variability; DT: discrimination thresholds; SDPE: standard deviation of wrist joint position error; JND: just noticeable difference threshold; VMI: visual motor integration; TVPS: test of visual perceptual skills; VD: visual discrimination; VM: visual memory; SR: spatial relationships; SM: sequential memory; VF: visual figure-ground; VC: visual closure; FC: form consistency; MFP: manual form perception; FI: finger identification; LTS: localization of tactile stimuli; DTSP: double tactile stimuli perception.

**Table 3:** Correlations between sensory processing impairment and motor coordination difficulties.

Scheme.	Sensory Process		Developmental Coordination Disorder Questionnaire (DCDQ)				Movement Assessment Battery for Children-2nd Edition (M-ABC2)			
			CDM	FM&M	GC	Total Score	MD	A&C	Bal	Total Score
1. Mikami et al., 2021 [23]	Auditory	DCD	-	-	−0.44 ***	-				
		TD	-	-	-	-				
	Touch	DCD	-	−0.34 **	-	-				
		TD	-	-	-	-				
	Multi-sensory	DCD	-	-	0.38 **	-				
		TD	-	-	−0.19 *					
	Sensation avoiding score	DCD	−0.42 ***	-	-	-				
		TD	-	-	-	-				
	Sensory sensitivity score	DCD	-	−0.34 **	-	-				
		TD	-	-	-	-				
	Sensation seeking score	DCD	0.32 **	-	-	-				
		TD	-	-	-	-				
	Low registration score	DCD	-	-	-	-				
		TD	-	-	−0.23 *	-				
2. Delgado-Lobete et al., 2020 [30]	Low registration score	DCD	-	-	-	-				
		TD	-	-	-	-				
		Both	−0.28 ***	-	−0.17 ***	−0.47 ***				
	Sensory sensitivity score	DCD	-	-	-	-				
		TD	-	-	-	-				
		Both	-	−0.14 ***	−0.16 ***	−0.29 **				
3. Nobusako et al., 2021 [31]	Visual (PSE)	DCD	NS	NS	NS	NS	NS	NS	NS	NS
		TD	-	-	-	-	-	-	-	-
		Both	-	-	-	-	0.43 **	-	-	-
4. Chen et al., 2020 [32]	Proprioceptive (PE) at the knee	DCD					NS	NS	–0.47 *	-
		TD					NS	NS	NS	-
	Proprioceptive (PE) at the ankle	DCD					NS	NS	–0.41 *	-
		TD					NS	NS	NS	-
	Proprioceptive (PEV) at the knee	DCD					NS	NS	–0.44 *	-
		TD					NS	NS	NS	-
	Proprioceptive (PEV) at the ankle	DCD					NS	NS	–0.42 *	-
		TD					NS	NS	NS	-
5. Tseng et al., 2019 [33]	Touch (discrimination thresholds)	DCD					-	-	-	-
		TD					-	-	-	-
		Both					−0.43 **	−0.40 **	−0.49 **	-
6. Tseng et al., 2018 [34]	Proprioceptive (SDPE)	DCD					-	-	-	-
		TD					-	-	-	-
		Both					NS	−0.29 *	−0.30 *	-
	Proprioceptive (JND thresholds)	DCD					-	-	-	-
		TD					-	-	–0.40 *	-
		Both					−0.40 **	NS	−0.50 ***	-
	Proprioceptive (PE)	DCD					-	-	-	-
		TD					-	-	-	-
		Both					NS	−0.08	NS	-
7. Prunty et al., 2016 [35]	Found no association between sensory and motor									
8. Cheng et al., 2014 [36]	Visual (discrimination)	DCD					NS	0.64 **	NS	NS
		TD					NS	NS	NS	NS
	Visual (Form constancy)	DCD					NS	NS	NS	NS
		TD					NS	NS	NS	NS
	Visual (Sequential memory)	DCD					0.65 **	NS	NS	NS
		TD					NS	NS	NS	NS
	Visual (Figure ground)	DCD					NS	NS	NS	NS
		TD					NS	NS	NS	NS
	Visual (Single in left—SL)	DCD					NS	NS	NS	–0.55 *
		TD					NS	NS	NS	NS
	Visual (Single in right—SR)	DCD					NS	NS	NS	NS
		TD					NS	NS	NS	NS
	Visual (double off in the left—OL)	DCD					NS	NS	NS	NS
		TD					NS	NS	NS	NS
	Visual (double off in the right—OR)	DCD					NS	−0.60 *	NS	–0.47 *
		TD					NS	NS	NS	NS
	Visual (double left—DL)	DCD					NS	−0.48 *	NS	NS
		TD					NS	NS	NS	NS
	Visual (double right—DR)	DCD					NS	–0.49 *	NS	NS
		TD					NS	NS	NS	NS
9. Cox et al., 2015 [37]	Touch (spatial tactile perception—SPL) in both hands	DCD					-	-	-	-
		TD					-	-	-	-
		Both					−0.04 **		1.44 **	1.39 **
10. Elbasan et al., 2012 [11]	Found no association between sensory and motor									

* *p* < 0.05, ** *p* < 0.01, *** *p* < 0.001; DCD, developmental coordination disorder; TD, typical development; Both: combining DCD children and TD children; (-): There is no correlation between sensory impairment and motor coordination problems; Blank square: not statistically analyzed in that area. NS, non-significant difference; CDM: control during movement; FM&H, fine motor/handwriting; GC, general coordination; MD, manual dexterity; A&C, aiming and catching; Bal: Bal.
